# Mesonephric-like adenocarcinoma of the uterine corpus: a case report

**DOI:** 10.3389/fmed.2026.1764322

**Published:** 2026-06-05

**Authors:** Ting Wang, Wenlin Yang, Xianglun Gong, Bing Xue

**Affiliations:** 1 Department of Pathology, Affiliated Hospital of Jining Medical University, Jining, China; 2 Department of Pathology, Nantong Tumor Hospital, Nantong, China; 3 Department of Pain, Jining No. 1 People’s Hospital, Jining, China

**Keywords:** endometrial carcinoma (EC), GATA3, mesonephric-like adenocarcinoma (MLA), uterine corpus carcinoma, immunohistochemistry

## Abstract

Mesonephric-like adenocarcinoma (MLA) is a rare and recently recognized subtype of endometrial and ovarian carcinoma characterized by distinctive morphology, immunophenotype, and aggressive clinical behavior. We report the case of a 60-year-old postmenopausal woman presenting with irregular vaginal bleeding. Imaging demonstrated a large uterine mass with deep myometrial invasion. Histopathological examination revealed diverse architectural patterns, including tubular, glandular, and slit-like structures, composed of cells with bland overlapping nuclei and occasional nuclear grooves. Immunohistochemical analysis showed positivity for GATA-3, TTF-1, EMA, and CK, and negativity for ER and PR, supporting the diagnosis of mesonephric-like adenocarcinoma of the uterine corpus.

## Introduction

Mesonephric adenocarcinoma (MA) is a rare malignancy of the female genital tract, frequently referred to as mesonephric-like adenocarcinoma (MLA) due to its unclear association with mesonephric remnants (1). While MLA typically arises in the uterine cervix and vagina, its occurrence in the uterine corpus is exceedingly rare, representing a distinct malignancy characterized by unique morphological, immunohistochemical, and molecular features. Here, we present a case of MLA originating in the uterine corpus.

## Case presentation

**FIGURE 1:**
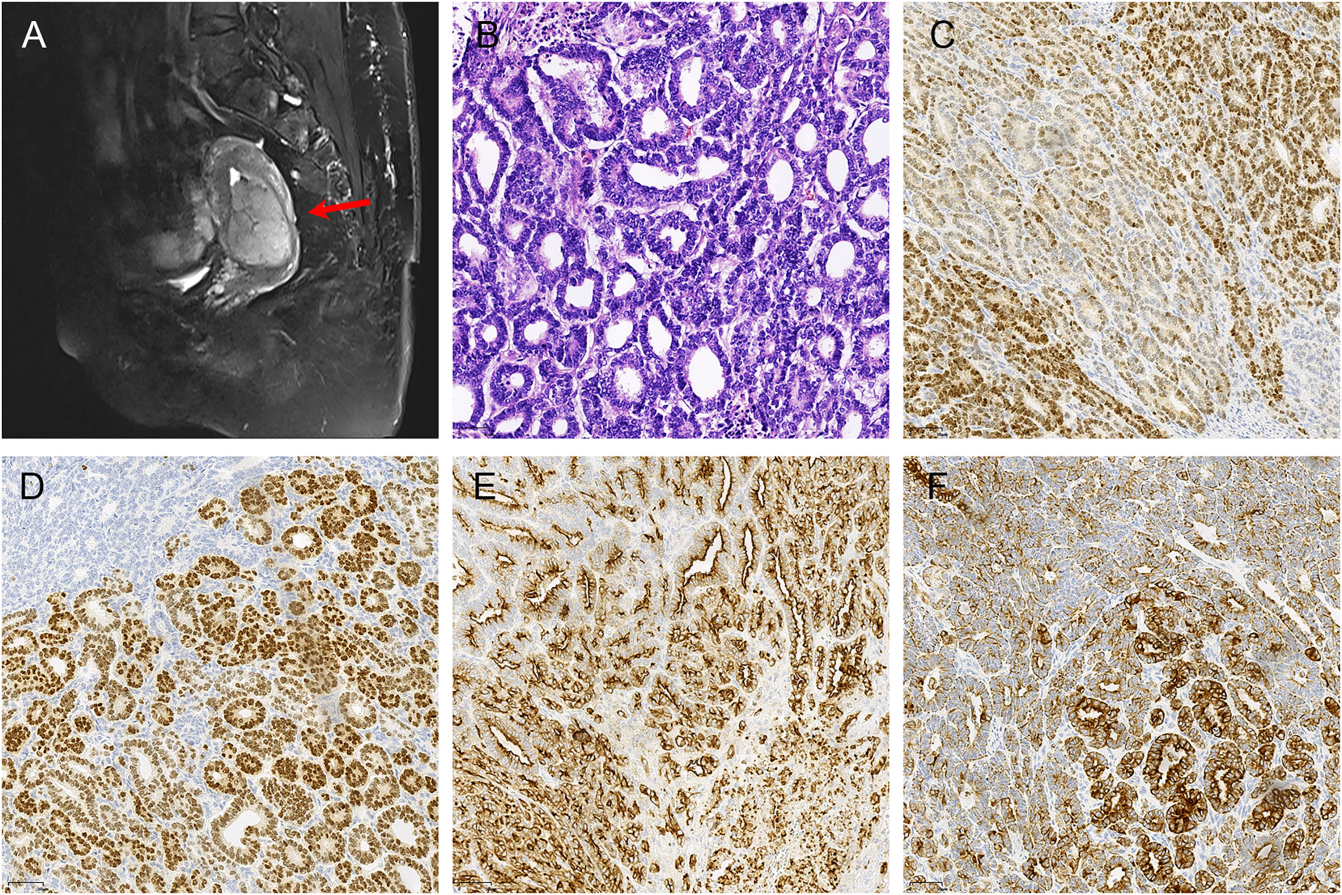
Radiologic and histopathological findings of mesonephric-like adenocarcinoma of the uterine corpus. **(A)** Sagittal T2-weighted MRI showing a large, exophytic polypoid mass occupying the uterine cavity and deeply invading the myometrium. **(B)** Higher-power view showing tumor cells arranged in tight glandular structures with crowded, overlapping nuclei (magnification, × 200). **(C)** Strong nuclear positivity for GATA-3 (magnification, × 100). **(D)** Strong nuclear positivity for TTF-1 (magnification, × 100). **(E)** Strong membranous and cytoplasmic positivity for EMA (magnification, × 100). **(F)** Strong cytoplasmic positivity for cytokeratin (CK) (magnification, × 100).

A 60-years-old postmenopausal woman with a 10-years history of menopause presented with irregular vaginal bleeding for 4 months. Transvaginal ultrasound revealed a hypoechoic mass in the lower uterine segment, and Magnetic Resonance Imaging (MRI) showed a thickened endometrium with a localized mass measuring approximately 30 mm × 34 mm × 52 mm, confined to the myometrium (Figure 1A). Laboratory tests for tumor markers (CA125, CA199, CEA, AFP, HE4, SCC) showed no significant abnormalities. Under general anesthesia, the patient underwent laparoscopic total hysterectomy, bilateral salpingo-oophorectomy, pelvic lymph node dissection, and para-aortic lymph node dissection. Intraoperative diagnosis confirmed endometrial carcinoma (FIGO stage T2N0M0).

Histological examination at high magnification (Figure 1B) revealed cuboidal to columnar cells with mild cytologic atypia, inconspicuous nucleoli, rare mitotic figures, scant cytoplasm, overlapping and crowded nuclei, and occasional nuclear grooves. Immunohistochemical analysis (Figures 1C–F) showed GATA-3 (+), TTF-1 (+), EMA (+), CK (+), Ki-67 index: ∼30%, Vimentin (partially+), PR (−), ER (−), Inhibin α (−), CD10 (−), P53 wild type (−), WT-1 (−). Special staining demonstrated reticulin fiber positivity. The patient was followed for 12 months and remained in good general health, with no recurrence or metastasis.

## Discussion

Mesonephric-like adenocarcinoma was first recognized as a distinct entity in the 2020 WHO Classification of Tumors of Female Reproductive Organs (2), where it was described as not being associated with embryonic mesonephric remnants. Uterine MLA is rare, but it is associated with aggressive biological behavior, including deeper myometrial invasion, more frequent cervical stromal extension, lymphovascular invasion, advanced stage, and higher rates of late recurrence and distant metastasis than MA (3). In addition to its distinctive morphologic and immunophenotypic features, mesonephric-like adenocarcinoma is increasingly characterized by recurrent molecular alterations. Previous studies have shown that activating mutations in *KRAS* are the most frequent genetic events in MLA, while additional alterations in genes such as *NRAS*, *PIK3CA*, *ARID1A*, and *PTEN* have also been reported (4). These molecular features support the unique pathogenesis of MLA and may aid in distinguishing it from other histologic mimics. Surgical resection remains the primary treatment for localized MLA. However, there is no established standard of post-operative therapy for uterine MLA. The most common adjuvant treatment is combination chemotherapy with carboplatin and paclitaxel. Due to the rarity of this malignancy and its potential for recurrence, long-term follow-up involving regular imaging (such as pelvic ultrasound, CT scans, or MRIs) and blood tests is crucial.

Because of the heterogeneous architecture of MLA, it is often misdiagnosed as other malignant neoplasms. The characteristic co-expression of GATA3 and TTF-1, along with negativity for ER and PR, supports mesonephric differentiation and helps distinguish MLA from endometrioid and serous carcinomas. By presenting this case, we aim to improve recognition of MLA in the uterine corpus. A multidisciplinary approach combining clinical evaluation, imaging, histopathology, immunohistochemistry, and molecular testing is essential for accurate diagnosis and optimal management.

## Data Availability

The original contributions presented in this study are included in this article/supplementary material, further inquiries can be directed to the corresponding authors.
